# Physiologically-Based Pharmacokinetics and Empirical Pharmacodynamic Modeling for Pediatric Henagliflozin Dosing: Clinical Insights for Chinese Patients

**DOI:** 10.1155/pedi/8857248

**Published:** 2025-08-07

**Authors:** Xinyue Zhang, Hao Xue, Jialei Xu, Ke Ren, Fangyi Qian, Yifan Zhang, Jingru Dou, Kai Shen, Xiao Zhu, Xiaoqiang Xiang, Qingfeng He

**Affiliations:** ^1^Department of Clinical Pharmacy and Pharmacy Administration, School of Pharmaceutical Sciences, Fudan University, Shanghai, China; ^2^Shanghai Institute of Materia Medica, Chinese Academy of Sciences, Shanghai, China; ^3^Department of Chemistry, University College London, London, WC1H OAJ, UK; ^4^Jiangsu Hengrui Medicine Co., Ltd., Shanghai, China; ^5^Quzhou Fudan Institute, Quzhou, China; ^6^State Key Laboratory of Advanced Drug Formulations for Overcoming Delivery Barriers, Shanghai, China

**Keywords:** Chinese population, henagliflozin, pediatric dosing, pharmacodynamic (PD) modeling, physiologically-based pharmacokinetic (PBPK) modeling

## Abstract

**Objective:** This study aimed to present a quantitative modeling and simulation approach for oral henagliflozin, a selective sodium-glucose cotransporter 2 (SGLT2) inhibitor primarily metabolized by uridine diphosphate-glucuronosyltransferase (UGT) enzymes.

**Methods:** A physiologically-based pharmacokinetic (PBPK) model for henagliflozin was developed using in vitro metabolism and clinical pharmacokinetic (PK) data, with validation across multiple contexts, including healthy adults, and hepatic impairment populations. Additionally, empirical pharmacodynamic (PD) modeling was employed to optimize pediatric dosing based on exposure-response relationships for urinary glucose excretion (UGE). Predicting henagliflozin exposure in pediatric patients poses challenges due to UGT enzyme ontogeny and the scarcity of clinical PK data in younger age groups. Using twofold acceptance criteria, model-predicted and observed drug exposures and PK parameters (area under the curve and peak concentration) were compared in diverse scenarios, including monotherapy in healthy adults (single/multiple doses), hepatic impairment, and extrapolation to pediatric age groups.

**Results:** The PBPK model accurately captured observed exposures within a twofold range in both adults and adolescents, supporting the model's predictive utility. The verified PBPK and empirical PD models informed dosing recommendations in pediatric populations aged 1 month to 18 years, achieving henagliflozin exposures comparable to those in adult patients receiving a 5–10 mg dose.

**Conclusion:** This study shows that PBPK and PD modeling effectively guide pediatric dosing of henagliflozin, reducing trial reliance and supporting real-world validation.

## 1. Introduction

**Figure 1:**
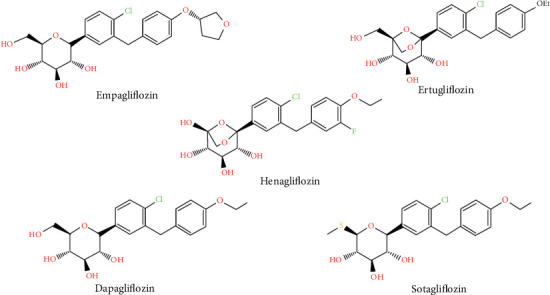
Structures for selected SGLT2 inhibitors.

Henagliflozin is a novel, orally administered, and highly selective sodium-glucose cotransporter 2 (SGLT2) inhibitor with over 800-fold greater selectivity for SGLT2 compared to SGLT1 [1]. It was developed and approved in China and shares structural similarities with other SGLT2 inhibitors like ertugliflozin and dapagliflozin (Figure 1) [2]. Clinical studies, including two phase 3 trials, have demonstrated that henagliflozin effectively improves glycemic control, reduces body weight, and lowers blood pressure in adult patients with type 2 diabetes mellitus (T2DM) when used as monotherapy at 5–10 mg once daily, or in combination with metformin or retagliptin [3–5].

The pharmacokinetic (PK) profile of henagliflozin in type 2 diabetic patients is consistent with that in healthy subjects. Following oral administration, henagliflozin reaches peak plasma concentration within 1.0–2.0 h, with peak concentration (*C*_max_) and area under the concentration–time curve (AUC) increasing proportionally to the dose. Food intake has no clinically significant impact on its absorption. Henagliflozin is highly bound to plasma proteins (94.5%−95.9%) and primarily metabolized by uridine 5'-diphospho-glucuronosyltransferase isoform 2B4/7, 1A9, 1A3, and 1A6 enzymes, forming three inactive glucuronide metabolites. It is predominantly excreted as metabolites in urine (30.1%) and unchanged in feces (50.0%), with an elimination half-life of 9.1–14.0 h and a total cumulative excretion rate of 80.1%. Figure S1 shows the henagliflozin disposition pathway based on absorption, distribution, metabolism, and excretion (ADME) studies with assumptions illustrated.

SGLT2 inhibitors, such as dapagliflozin and empagliflozin, are established treatments for adults with T2DM, chronic kidney disease (CKD), and heart failure [6]. While SGLT2 inhibitors are well-established in adults, their translation to pediatrics is delayed by developmental maturation of drug metabolizing enzymes, resulting in age-dependent PKs that preclude simple dose scaling from adult data [7]. Recently, empagliflozin (marketed as Jardiance) received Food and Drug Administration (FDA) approval for pediatric use in children 10 years and older based on positive results from the DINAMO phase 3 clinical trial [8]. Despite this advancement, other SGLT2 inhibitors, including henagliflozin remain uncertain in pediatric care, primarily due to the lack of robust clinical data [9]. However, clinical trials for these populations often face challenges such as ethical concerns, recruitment difficulties, and the need for age-appropriate formulations. To date, no clinical trials for henagliflozin have enrolled participants under 18 years of age, and registrational studies explicitly exclude pediatric populations. These gaps indicate the need for quantitative modeling approaches to inform pediatric dosing strategies across a full age spectrum, especially for adolescents with increased risks of T2DM.

Fortunately, physiologically-based PK (PBPK) modeling has emerged as a valuable and recognized tool for overcoming these challenges [10]. By integrating age-specific physiological parameters, PBPK models enable the prediction of drug PKs across different subpopulations, including geriatrics and pediatrics. This approach allows for the simulation of various dosing scenarios, accounting for ontogeny and population variability without exposing children to unnecessary risks [11]. Numerous PBPK models have been successfully verified to reproduce adult clinical PK profiles with high reliability. These verified models can be further leveraged to predict drug PK in pediatric populations when direct clinical study data is unavailable [12–14]. By incorporating pediatric-specific physiological factors, such as enzyme ontogeny, body weight, and renal excretion maturity, PBPK models offer a framework to predict age-appropriate drug exposure, optimize dosing strategies, and support clinical decision-making in children [15, 16]. In recent years, the U.S. FDA, the European Medicines Agency (EMA), and the International Council for Harmonization of Technical Requirements for Pharmaceuticals for Human Use (ICH) have released guidelines supporting pediatric extrapolation and advocating for the use of modeling and simulation to inform pediatric drug development and optimize dose selection [17, 18].

This study aims to develop and validate a PBPK model for henagliflozin to predict its PK in special populations. By leveraging an established adult PBPK model, we seek to simulate age-appropriate dosing regimens for pediatric populations, considering developmental physiological changes. The objective is to provide a scientific foundation for optimal pediatric dosing, thereby reducing the need for extensive pediatric trials and supporting regulatory and clinical decision-making for henagliflozin's use in pediatric patients with T2DM.

## 2. Materials and Methods

### 2.1. Software and Tools

In this study, the PBPK model development was conducted using the Open Systems Pharmacology Suite, incorporating PK-Sim (Version 11, Open Systems Pharmacology Suite, https://www.open-systems-pharmacology.org/). PK parameters and statistical analyses were performed using R software (Version 4.0, https://www.r-project.org/) and graphical visualizations were created using GraphPad Prism (Version 8.0, https://www.graphpad.com/). Physicochemical properties were retrieved using the DrugBank database (https://go.drugbank.com/drugs/DB11939), PubChem database (https://pubchem.ncbi.nlm.nih.gov/compound/Henagliflozin), and related literature sources. PK data were collected by searching literature databases, including PubMed and China National Knowledge Infrastructure (CNKI). Plasma drug concentration–time curve data were digitized using the GetData Graph Digitizer (Version 2.22, http://www.getdata-graph-digitizer.com/).

### 2.2. Development and Evaluation of Adult PBPK Model

#### 2.2.1. Model Structure and Parameterization

**Figure 2:**
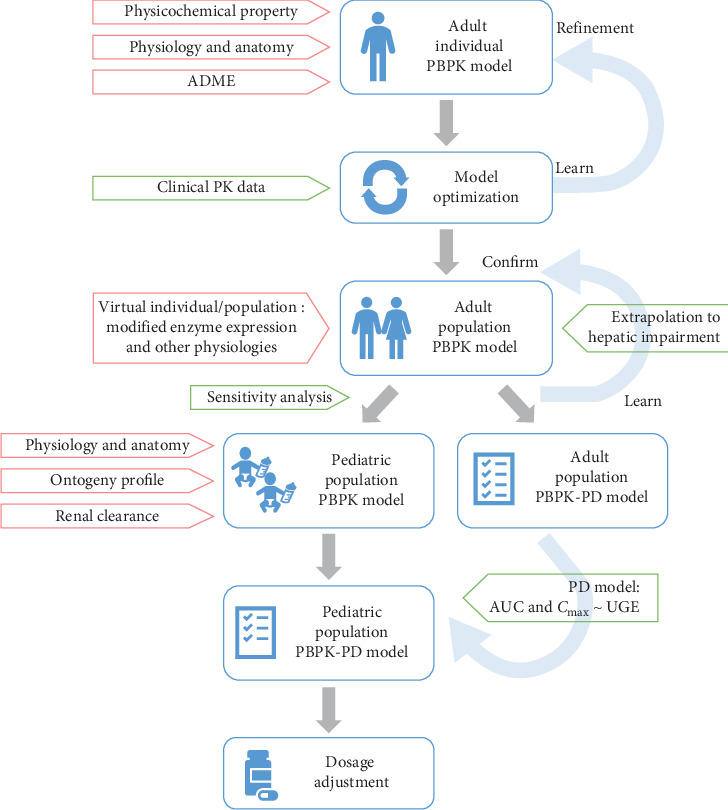
The workflow of PBPK model in adult and pediatric populations for henagliflozin.

A combination of “top-down” and “bottom-up” modeling strategies was applied to develop the models. The modeling workflow is illustrated in Figure 2. A whole-body, 18-compartment PBPK model was developed using PK-Sim for modeling and simulation. Each compartment consisted of four subcompartments: blood, plasma, interstitial, and intracellular space [19]. A virtual individual representing a typical 30-year-old male of East Asian ethnicity was created using average physiological parameters, and the virtual population of 100 individuals was generated based on this profile using the “population” building block in the software. Each population consisted of 50 males and 50 females within the age range of 18–45 years. The adult model for henagliflozin was developed using collected physicochemical properties, in vitro characteristics, and in vivo ADME parameters. Initial parameters were optimized using the Monte Carlo algorithm, with detailed parameter information provided in Table 1. All dosing protocols corresponding to each clinical study were accurately reproduced within the “administration protocol” building block of PK-Sim, ensuring that dosing regimens, including both single and multiple doses under fasted or fed states, reflected the clinical conditions without altering other parameters. Additionally, a “feeding event” was incorporated in PK-Sim using a default 1000 kcal meal to simulate the impact of food on PK.

Henagliflozin is structurally similar to the SGLT2 inhibitor ertugliflozin, sharing a comparable metabolic pathway (Figure S1) [22]. In humans, two glucuronide metabolites of ertugliflozin have been identified: a minor metabolite, ertugliflozin 2-O-glucuronide (mediated by UGT2B7/2B4), and a major one, ertugliflozin 3-O-glucuronide (mediated by UGT1A9) [23]. Based on human mass balance studies, ertugliflozin shows significant oxidative metabolism (fCL = 0.12), with systemic clearance largely mediated by UGT1A9 (70%), UGT2B7/2B4 (16%), CYP3A4 (10%), CYP3A5 (1.2%), CYP2C8 (0.5%), and renal elimination (2%). Similarly, data from in vitro studies and human mass balance for henagliflozin suggest that its clearance is primarily through UGT1A9 (47%), UGT2B7/2B4 (30%), and UGT1A3 (9%), with a smaller contribution from CYP enzymes (11%) [24, 25]. Approximately 1.9%–3% of henagliflozin is cleared renally as the unchanged drug [26, 27]. To assign enzymatic clearance rates in PK-Sim, a retrograde translation strategy was employed, using these fraction metabolized (fm) values to calculate intrinsic clearance (CL_int_) for each enzyme. In vitro studies with human liver and kidney microsomes indicated that 10% of UGT clearance occurs in the kidneys, and 90% in the liver [23]. For modeling, metabolism by UGT2B4/2B7 was attributed to UGT2B7, and CYP3A4 represented the CYP family, with optimized abundance in the PBPK model. Initial input parameters for henagliflozin were set to UGT1A9 CL_int_ at 21.6 µL/min/mg, UGT2B7 CL_int_ at 13.8 µL/min/mg, UGT1A3 CL_int_ at 4.1 µL/min/mg, and CYP family CL_int_ at 0.05 µL/min/pmol.

#### 2.2.2. Model Optimization and Validation

Clinical PK data for oral administration of henagliflozin in healthy subjects were obtained from the clinical research, as detailed in Table S1. These training dataset encompassed a range of single and multiple dosing regimens, with doses from 1.25 to 200 mg, administered both as single doses and daily over multiple days [26]. Validation of the model relied on studies from others involving healthy individuals under both fasting and fed conditions, receiving 5–25 mg single and multiple doses [25, 28–33]. In addition to healthy subjects, the model was also evaluated using data from studies involving patients with T2DM [34]. Since T2DM has been reported to have no significant impact on the PK of henagliflozin, no additional adjustments were made for the disease (except the age range for the virtual population was modified to 45–60 to match clinical demographics). The same model structure and parameterization applied to healthy subjects were used for T2DM patients, ensuring consistent evaluation of PK parameters. The geometric mean of the predicted plasma concentrations as well as urine excretion profile (if available) was obtained through software simulation. Time-concentration curves predicted by the model were visually compared to the observed clinical data. Model performance was evaluated by comparing predicted and observed PK parameters with goodness-of-fit plots, including *C*_max_ and AUC for single-dose administration, as well as steady-state *C*_max_ (*C*_max,ss_) and AUC (AUC_ss_) for multiple-dose administration. The fold error (FE) was calculated, while the average FE (AFE) and absolute AFE (AAFE) were computed for all plasma concentrations to quantify prediction accuracy (Equations (1)–(3)). Predictive performance was considered acceptable when these values were within a twofold range (0.5–2), and a stricter 1.25-fold limit (0.8–1.25) indicated higher predictive accuracy. Additionally, average absolute prediction error (AAPE) was calculated to measure prediction error scaled to percentage units (Equation (4)). A model is deemed satisfactory if the AAPE is less than 20%, acceptable if the AAPE is between 20% and 50%, and poor if the AAPE is 50% or higher [35].

(1) $$\begin{array}{c} \text{FE}=\;\frac{\text{Predicted value}}{\text{Observed value}}. \end{array}$$

(2) $$\begin{array}{c} \text{AFE}=\;10^{\frac{1}{N}\sum_{i=1}^{N}\log_{10}\left(\frac{{\text{Predicted}}_{i}}{{\text{Observed}}_{i}}\right)}. \end{array}$$

(3) $$\begin{array}{c} \text{AAFE}=\;10^{\frac{1}{N}\sum_{i=1}^{N}\left|\log_{10}\left(\frac{{\text{Predicted}}_{i}}{{\text{Observed}}_{i}}\right)\right|}. \end{array}$$

(4) $$\begin{array}{c} \text{AAPE}=\text{ Average }\left(\left|\frac{{\text{Predicted}}_{i}-{\text{Observed}}_{i}}{{\text{Observed}}_{i}}\right|\right). \end{array}$$

### 2.3. Extrapolation to Hepatic Impaired PBPK Model and Evaluation

The healthy adult PBPK model was extrapolated to simulate PK in populations with hepatic impairment. For the simulations, virtual individuals and populations (*n* = 100) were created with demographic characteristics aligned with clinical study data [36]. Patients were categorized based on the child-pugh classification: child-pugh A (CP-A), child-pugh B (CP-B), and child-pugh C (CP-C). Hepatic impairment leads to significant morphological and functional changes, including reduced hepatocyte function, altered hemodynamics, decreased plasma protein levels, and compromised renal function. These changes influence drug behavior and necessitate adjustments to enzyme expression abundance and other physiological factors. Several physiological changes were adapted for hepatic impairment, including reduced portal and renal blood flow, increased liver blood flow, and adjusted organ flows except for the brain [37, 38] (Table 2). Specifically, the expression of key metabolic enzymes, such as UGT1A9, UGT2B4/7, and CYP3A4, which play a major role in henagliflozin clearance, were reduced [39]. Additionally, the variability of plasma protein scale factor in PK-Sim was defined to reflect changes in plasma protein concentration and binding among populations [40]. These adjustments were made to capture the impact of hepatic impairment on PK, and simulations were performed for 20 mg single-dose administration. Model performance was evaluated by comparing predicted and observed plasma concentration-time profiles and key PK parameters like *C*_max_ and AUC across healthy controls and hepatic-impaired populations [36].

### 2.4. Extrapolation to Pediatric PBPK Model and Dose Optimization

Age-related physiological and anatomical parameters, such as height, weight, organ volume, and blood flow, were scaled using the built-in algorithms of PK-Sim. The ontogeny of key metabolic enzymes involved in henagliflozin clearance, including UGT1A9, UGT2B7, and CYP3A4, was integrated into the pediatric PBPK model using built-in ontogeny database in PK-Sim (Figure S2) [41]. Additionally, key parameters for clearance, including unbound fraction, GFR, and enzyme ontogeny factors, were adjusted to reflect pediatric physiology. Four virtual pediatric subgroups were created based on the ICH classification of pediatrics: neonates (0–27 days), infants and toddlers (28 days–23 months), children (2–11 years), and adolescents (12–18 years) [42]. Each subgroup consisted of 100 virtual individuals (50% female).

Given the lack of clinical data for validation, a sensitivity analysis was performed to evaluate the impact of key physiological parameters on henagliflozin's PKs in pediatric populations. Parameters such as enzyme activity (UGT1A9 and UGT2B7), renal function (GFR), and other physiological parameters were varied within plausible ranges to assess their influence on AUC and *C*_max_ [13]. Both local and global sensitivity analyses were conducted. Local sensitivity analysis (LSA) evaluated the impact of individual parameters (e.g., UGT enzyme activity and GFR) on AUC and *C*_max_ using a one-at-a-time perturbation approach. In addition, global sensitivity analysis (GSA) was performed using Morris screening and extended Fourier amplitude sensitivity test (EFAST) methods to account for parameter uncertainty, nonlinearity, and potential interactions [43, 44]. Full methodological details and sensitivity index values are presented in Table S2.

Pediatric doses were optimized to achieve a systemic exposure comparable to adults. To further optimize pediatric doses and ensure clinical efficacy, a pharmacodynamic (PD) model was integrated into the pediatric PBPK framework. Previously reported exposure-response relationships, as shown in the following equations (Equations (5) and (6)), were utilized to link henagliflozin doses and exposure to therapeutic outcomes, particularly focusing on urinary glucose excretion (UGE) [26].

(5) $$\begin{array}{c} E=\frac{E_{\max}\times C_{\max}}{{\text{EC}}_{50}+C_{\max}}, \end{array}$$

(6) $$\begin{array}{c} E=\frac{E_{\max}\times \text{AUC}}{{\text{EC}}_{50}+\text{AUC}}, \end{array}$$

where *E* is the effect (UGE, g), *C*_max_ is the maximum concentration of the drug in plasma, AUC is the area under the concentration–time curve, and EC_50_ is the concentration or AUC at which 50% of the maximum effect is achieved. Based on the results from clinical studies, *E*_max_ and EC_50_ were determined to be 48.8 g and 62.0 ng/mL for *C*_max_ based relationships, respectively. For AUC based equation, *E*_max_ and EC_50_ were determined to be 48.2 g and 356 ng h/mL, respectively.

## 3. Results

### 3.1. Adult PBPK Model Development and Validation

**Figure 3:**
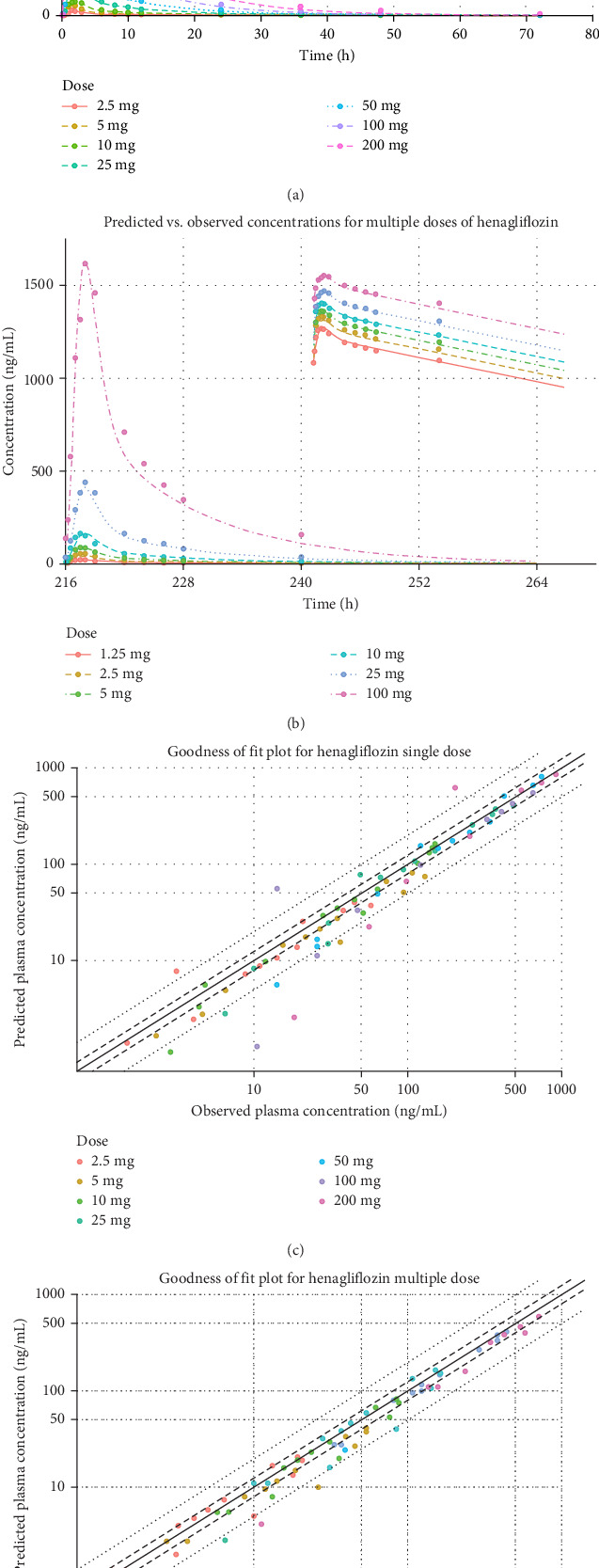
Predicted vs. observed plasma concentrations and goodness of hit for henagliflozin at different doses. (A) Predicted vs. observed plasma concentrations for different single doses of henagliflozin ranging from 2.5 to 200 mg, with log–log scaling. (B) Predicted vs. observed plasma concentrations for different multiple doses of henagliflozin ranging from 1.25 to 100 mg, with log–log scaling. (C) Goodness of fit plot for henagliflozin single-dose predictions. (D) Goodness of fit plot for henagliflozin multiple-dose predictions. The solid line represents the 1.25-fold error, and the dashed lines represent the twofold error boundaries, for evaluating prediction performance.

**Figure 4:**
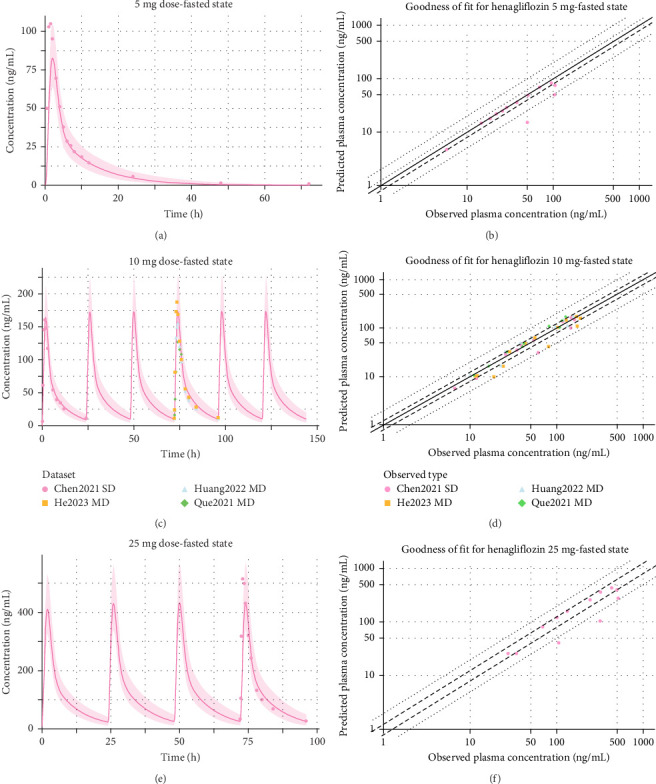
Predicted vs. observed plasma concentrations and goodness of fit for henagliflozin under fasted state at various doses. (A) Predicted vs. observed plasma concentrations for a 5 mg dose, fasted state. (C) Predicted vs. observed concentrations for a 10 mg dose, fasted state, with repeated dosing. (E) Predicted vs. observed concentrations for a 25 mg dose, fasted state, with repeated dosing. Shaded areas represent 5%–95% prediction intervals, solid lines show model predictions, and symbols indicate observed data points from different datasets. (B), (D), and (F) present GOF plots for 5, 10, and 25 mg doses, respectively, in the fasted state. Solid lines represent the 1.25-fold error, while dashed lines represent the twofold error boundaries, providing an assessment of model prediction accuracy.

**Figure 5:**
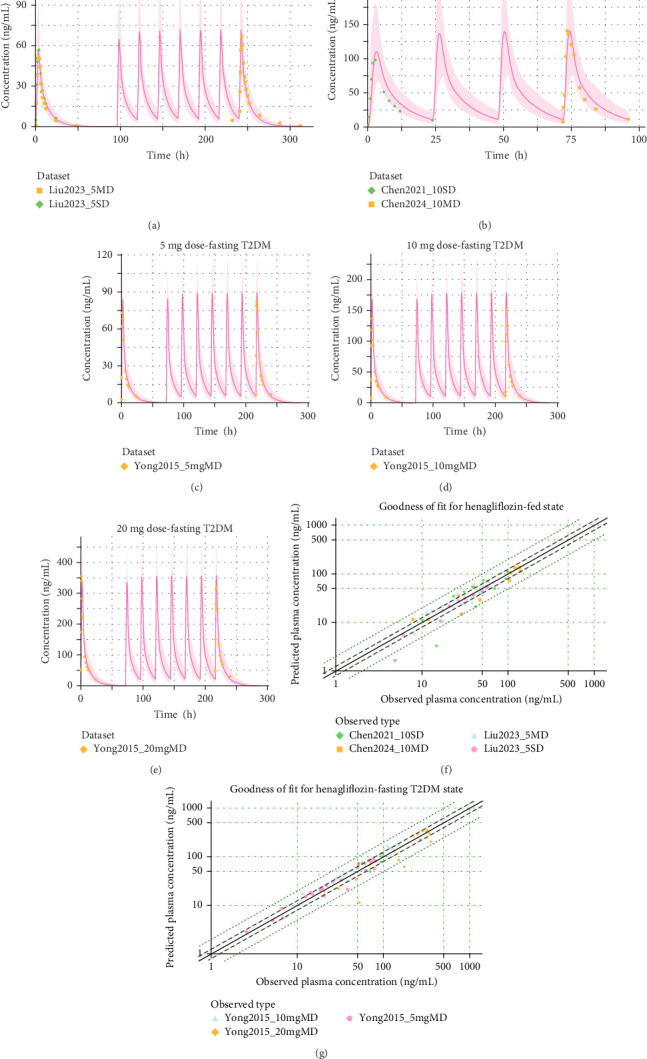
Predicted vs. observed plasma concentrations and goodness of fit for henagliflozin under fed and fasting dtates in T2DM Patients. (A, B) show predicted vs. observed plasma concentrations for 5 and 10 mg doses in the fed state, respectively, while (C–E) display 5, 10, and 20 mg doses under fasting conditions in T2DM patients. The shaded areas represent 5%–95% prediction intervals, with solid lines indicating model predictions and symbols representing observed data points. (F, G) illustrate the GOF plots for the fed and fasting T2DM states, respectively, with solid lines representing the 1.25-fold error and dashed lines indicating the twofold error boundaries for performance assessment.

The henagliflozin PBPK model was successfully developed for adults and validated using clinical data from both healthy individuals and patients with T2DM. Simulated PK profiles closely matched the observed data, with predicted values for *C*_max_ and AUC (and fraction of dose in urine in the training datasets) within a two-FE range for FE and less than 50% for AAPE (Table 3), which summarizes all PK comparisons. GOF plots further demonstrated strong concordance between predicted and observed plasma concentrations across all single and multiple dose regimens, with Figure 3 displaying results for the training dataset and Figures 4 and 5 for the validation dataset.

### 3.2. Validation Through Extrapolation to Hepatic Impaired Populations

The henagliflozin PBPK model was extrapolated to populations with varying degrees of hepatic impairment (CP-A, CP-B, and CP-C) and compared against a control group of healthy individuals. Performance indicators, including AFE, AAFE, and AAPE, were calculated for both AUC_0–72_ and *C*_max_, providing insights into the model's predictive accuracy across different impairment levels (Table 4 and Figure S3). In the control group, the model performed well, with an AFE of 1.15 for AUC and 1.04 for *C*_max_, both within an acceptable range. Predictions in CP-A and CP-B groups showed slightly higher errors, with AAFE values for AUC ranging from 1.24 to 1.61, while *C*_max_ errors remained moderate. However, in the CP-C group, representing severe hepatic impairment, the model showed a larger deviation in AUC predictions, with an AAPE of 27.60%, indicating increased variability in this population. Despite these variations, the overall performance remains consistent within an acceptable range for clinical application, suggesting the model's utility in predicting henagliflozin exposure in hepatic impaired populations.

### 3.3. Extrapolation to Pediatric Populations

The PBPK model predictions for henagliflozin across various pediatric subgroups (neonates, infants/toddlers, children, and adolescents) were evaluated in terms of *C*_max,ss_, AUC_ss_, and corresponding UGE. As shown in Table 5, the predicted *C*_max_ and AUC values increased with higher doses in all age groups, consistent with the dose–exposure relationship observed in adults. However, younger age groups, particularly neonates and infants, exhibited disproportionately higher *C*_max,ss_ and AUC_ss_ values compared to older pediatric populations at equivalent doses. The UGE results demonstrated a dose-dependent increase across all pediatric age groups.

**Figure 6:**
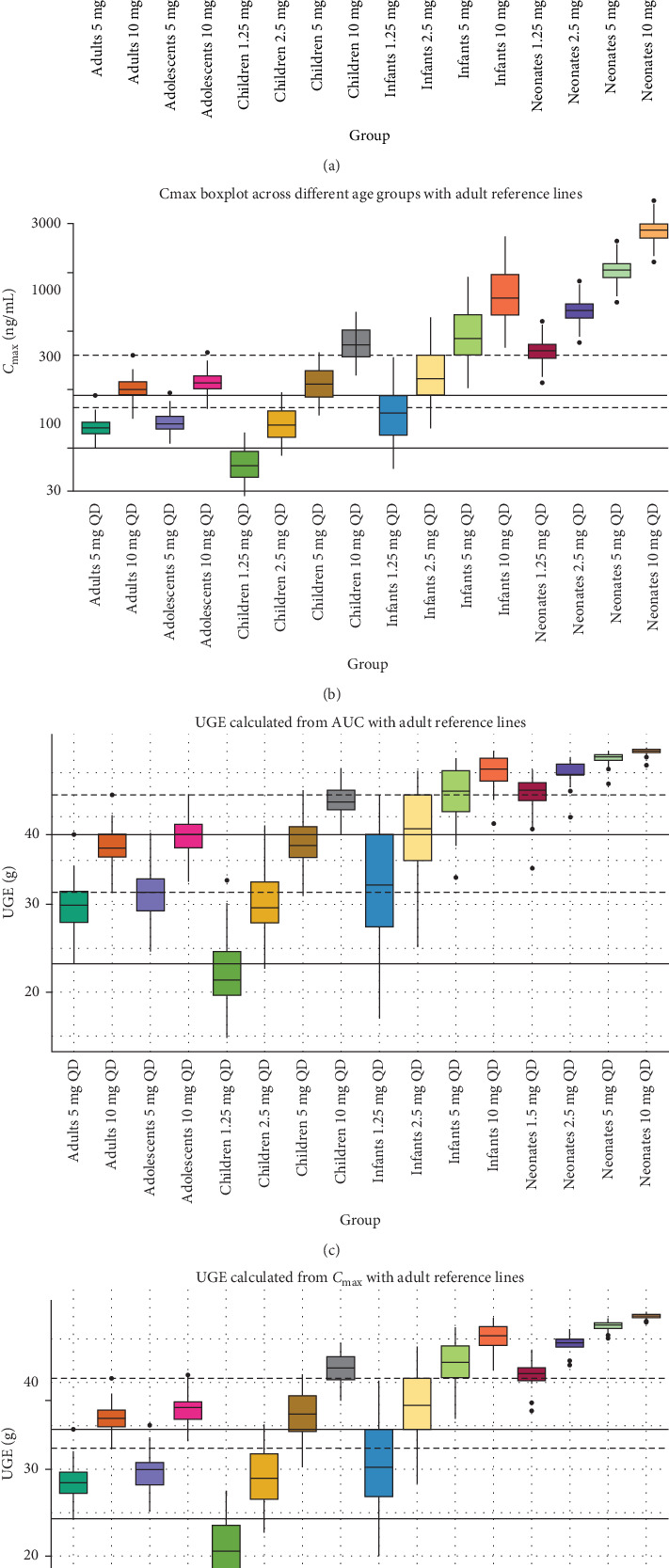
Boxplots of AUC, *C*_max_, and UGE across different age groups with adult reference lines. (A) AUC values across age groups from neonates to adults, with adult 5 and 10 mg reference ranges indicated by solid and dashed lines, respectively. (B) *C*_max_ values across age groups, with the solid line representing the adult 5 mg reference range and the dashed line indicating the adult 10 mg range. (C) UGE calculated from AUC values, with reference lines as in (A). (D) UGE calculated from *C*_max_ values, with reference lines as in (B).

Based on the exposure and UGE results (Table 5 and Figure 6), pediatric dosing recommendations can be made by comparing to adult exposures at 5 and 10 mg once daily. In adolescents (12–18 years), 5 and 10 mg doses achieve *C*_max_, AUC, and UGE levels comparable to adults, suggesting adult doses can be applied directly. For children (2–11 years), 2.5 mg corresponds to adult-equivalent exposure for 5 and 5 mg corresponds to adult-equivalent exposure for 10 mg. In infants and toddlers (28 days–23 months), 1.25 mg produces exposure and UGE values similar to adults on 5 mg, while 2.5 mg aligns with adult 10 mg doses. For neonates (0–27 days), 1.25 mg provides exposures comparable to adults on 10 mg, suggesting lower doses are needed. These findings support tailored pediatric dosing that achieves adult-equivalent exposure and therapeutic outcomes.

The sensitivity analysis showed that the pediatric PBPK model was generally robust, with most input parameters having relatively low sensitivity scores for both AUC and *C*_max_ (Figure S4). Parameters such as UGT ontogeny factors, kidney GFR (specific), and gastrointestinal transit times had slightly more pronounced effects, but their overall influence on the model outputs was still moderate, with negligible interaction effects between parameters (Figure S5). This suggests that the extrapolation of adult data to pediatric populations, based on the current model, is solid and reliable, with minimal impact from small variations in individual physiological parameters. Thus, the model is suitable for pediatric dose optimization and provides confidence in the proposed dosing recommendations.

## 4. Discussion

This study demonstrates the utility of PBPK and empirical PD modeling in addressing the unique challenges of Chinese pediatric dosing for henagliflozin, a locally developed SGLT2 inhibitor. The Research to Accelerate Cures and Equity for Children Act, along with FDA guidance, further emphasizes the inclusion of children in clinical trials when possible to improve the availability of pediatric-specific safety and efficacy data [45]. These initiatives highlight the need for early PK predictions to ensure appropriate and safe exposure levels during pediatric studies. Modeling and simulation have been instrumental in expanding the use of other SGLT2 inhibitors, such as ertugliflozin and dapagliflozin, to pediatric populations [46, 47]. Although neither drug was originally intended for pediatric populations, pharmacometric models have facilitated their adaptation for younger age groups by incorporating developmental PK considerations, including age-related changes in enzyme abundance and activity. For SGLT2 inhibitor uses in pediatric populations, age-related maturation of physiological processes, particularly the ontogeny of enzymes like UGT1A9 and UGT2B7, plays a crucial role in drug metabolism and disposition. Young children, particularly those aged 2–11 years old, have altered maturation of these physiological factors, impacting ADME and ultimately affecting drug efficacy and safety.

Importantly, this study's focus on Chinese pediatric patients fills a significant gap in current SGLT2 inhibitor research, where much of the available data are based on Asian adult or non-Asian pediatric populations. Following the successful modeling approaches used for henagliflozin's analogs (ertugliflozin and dapagliflozin), our study leveraged available adolescent data to refine a PBPK model for henagliflozin, incorporating age-specific physiological and metabolic parameters. Our model addressed these developmental differences by incorporating age-specific physiological parameters, which were further refined through sensitivity analyses. By validating this model against clinical data from healthy adults and patients with hepatic impairment, we confirmed its predictive accuracy and built a foundation for henagliflozin exposure predictions in pediatric subgroups. The model integrates enzyme ontogeny profiles to reflect age-dependent metabolic activity and proposes initial dosing recommendations for pediatric patients that achieve systemic exposure levels comparable to a 5–10 mg adult dose. The empirical PD model further complemented the PBPK framework by linking dose-exposure relationships to therapeutic outcomes, focusing on UGE as a key PD marker. In adolescents (12–18 years), 5 and 10 mg doses achieve comparable PK and PD levels to adults, suggesting adult doses can be applied directly. For children (2–11 years), 2.5 mg corresponds to adult-equivalent exposure for 5 and 5 mg corresponds to adult-equivalent exposure for 10 mg. The combined PBPK and PD models thus offer a robust approach to establishing clinically meaningful dose adjustments for children at various developmental stages, supporting the growing interest in applying quantitative pharmacology to personalize dosing regimens in China, particularly for drugs developed domestically, like henagliflozin.

A significant aspect of this study is the broader applicability of SGLT2 inhibitors beyond T2DM, as recent studies highlight their benefits in managing CKD and congestive heart failure (CHF) across adult populations [48, 49]. These additional indications, alongside the growing interest in pediatric treatment options, underscore the need for age-appropriate dosing of SGLT2 inhibitors in pediatric populations with complex health profiles. Pediatric CKD and CHF represent significant unmet needs, where safe and effective drug exposures are essential yet challenging to establish due to age-related physiological changes [9]. Our PBPK and PD models provide a foundation for these broader applications by predicting dose–response relationships in pediatric populations, potentially guiding safe and effective dosing in CKD and CHF pediatric patients as further clinical evidence becomes available.

This study has limitations that warrant consideration. While hepatic modeling was primarily used for adult validation, its ability to capture reduced enzyme activity supports its relevance for pediatric extrapolation, where enzyme maturation similarly influences clearance. Nonetheless, due to the lack of direct clinical data in neonates and infants, predictions in these subgroups should be interpreted cautiously. The model currently relies on standard ontogeny functions, which may not fully capture age-specific variability. More refined quantitative data, including sex-specific enzyme expression during adolescence, could improve future predictions. On the PD side, our model assumes similar UGE relationships across ages, though renal glucose thresholds may vary. Future studies collecting pediatric UGE or HbA1c data are needed to refine the PD model [20]. Additionally, incorporating kidney-specific exposure and absorption variability, particularly in infants, would enhance mechanistic insights. We also recommend real-world pediatric studies to externally validate and adjust dosing strategies, especially as safety and effectiveness data emerge in broader clinical settings.

## 5. Conclusion

In summary, this study demonstrates the effectiveness of integrating PBPK and empirical PD modeling to inform pediatric dosing strategies for henagliflozin, with a focus on the Chinese population. Our modeling approach successfully predicts age-appropriate dosing regimens, providing a science-based framework that minimizes the need for extensive pediatric trials. Future work incorporating real-world pediatric data and mechanistic refinements will further enhance the accuracy and clinical relevance of this model.

## Figures and Tables

**Table 1:** Input parameters for developing the henagliflozin PBPK model.

Parameter	Initial value	Reference	Final value	Source
Physiochemical properties/formulation				
LogP	1.8–2.46	DrugBank; PubChem	2.1	Optimized
MW (g/mol)	454.9	DrugBank; PubChem	454.9	—
Solubility (mg/mL)	0.182	DrugBank	0.182	—
Dissolution time (50% dissolved, min)	240	Default	87	Optimized
Dissolution shape	0.92	Default	1.70	Optimized
Absorption				
Specific intestinal permeability (cm/s)	6.7 × 10^−4^	[20]	6.7 × 10^−4^	—
Distribution				
*f*_u_	5%	[20]	5%	—
*B*/*P* ratio	0.55		0.55	—
Partition coefficients	PK-Sim standard	Default	Poulin and Theil	Optimized
Cellular permeabilities	PK-Sim standard	Default	PK-Sim standard	Default
Metabolism				
CL_int,CYP_	0.05 µL/min/pmol rec. enzyme	Calculated	0.08 µL/min/pmol	Optimized
CL_int,UGT1A9_	21.6 µL/min/mg	Calculated	32.4 µL/min/mg	Optimized
Content in liver microsomes	61.2 pmol/mg mic. protein	[21]	61.2 pmol/mg mic. protein	—
CL_int,UGT2B7_	13.8 µL/min/mg	Calculated	20.7 µL/min/mg	Optimized
Content in liver microsomes	200 pmol/mg mic. protein	[21]	200 pmol/mg mic. protein	—
CL_int,UGT1A3_	4.1 µL/min/mg	Calculated	6.3 µL/min/mg	Optimized
Content in liver microsomes	20.6 pmol/mg mic. protein	[21]	20.6 pmol/mg mic. protein	—
Elimination				
GFR fraction	1	Default	0.70	Optimized

Abbreviations: *B*/*P* ratio, blood/plasma ratio; CL_int_, intrinsic clearance; CYP, cytochrome P450; *f*_u_, fraction unbound; GFR, glomerular filtration rate; LogP, lipophilicity; MW, molecular weight.

**Table 2:** Physiological changes associated with hepatic impairment.

Parameter	Healthy	CP-A	CP-B	CP-C
Blood flow rate fraction				
Portal	1	0.40	0.36	0.04
Hepatic arterial	1	1.30	2.30	3.40
Renal	1	0.88	0.65	0.48
Brain	1	1	1	1
Other organs	1	1.75	2.25	2.75
Liver volume fraction	1	0.69	0.55	0.28
Hematocrit value	0.47	0.39	0.37	0.35
Ontogeny factor (albumin)	1	0.81	0.68	0.50
Ontogeny factor (α1-acid glycoprotein)	1	0.60	0.56	0.30
Enzyme reference concentration				
UGT1A9 (μmol/L)	1	1	0.48	0.28
UGT2B7 (μmol/L)	1	1	0.30	0.13
CYP3A4 (μmol/L)	4.32	4.32	0.86	0.86
GFR fraction	1	1	0.70	0.36

Abbreviations: CP-A/B/C, chil-pugh A/B/C; GFR, glomerular filtration rate.

**Table 3:** Prediction performance for henagliflozin PBPK model in adults.

Group	Parameter	AFE	AAFE^a^	AAPE%
Training dataset: fasting, healthy, single dose				
2.5 mg	AUC_0–24_	0.79	0.79	21.3
	*C*_max_	0.70	0.70	29.6
	*A*_e_	0.70	0.70	0.30
5 mg	AUC_0–24_	0.77	0.77	22.7
	*C*_max_	0.63	0.63	37.2
	*A*_e_	0.58	0.58	0.42
10 mg	AUC_0–24_	0.94	0.94	6.2
	*C*_max_	1.08	1.08	7.6
	*A*_e_	0.69	0.69	0.31
25 mg	AUC_0–24_	0.93	0.93	7.4
	*C*_max_	0.83	0.83	16.7
	*A*_e_	0.71	0.71	0.29
50 mg	AUC_0–24_	0.90	0.90	10.3
	*C*_max_	0.77	0.77	22.8
	*A*_e_	0.65	0.65	0.35
100 mg	AUC_0–24_	0.96	0.96	4.3
	*C*_max_	1.11	1.11	11.0
	*A*_e_	0.67	0.67	0.33
200 mg	AUC_0–24_	0.94	0.94	6.1
	*C*_max_	0.96	0.96	4.4
	*A*_e_	0.65	0.65	0.35
Training dataset: fasting, healthy, multiple dose				
1.25 mg	AUC_ss_	1.07	1.07	7.28
	*C*_max,ss_	0.99	0.99	1.19
	*A*_e_	0.91	0.91	0.09
2.5 mg	AUC_ss_	0.77	0.77	23.15
	*C*_max,ss_	0.75	0.75	24.51
	*A*_e_	0.72	0.72	0.28
5 mg	AUC_ss_	0.93	0.93	6.79
	*C*_max,ss_	0.93	0.93	6.68
	*A*_e_	0.88	0.88	0.13
10 mg	AUC_ss_	1.03	1.03	3.20
	*C*_max,ss_	1.01	1.01	0.54
	*A*_e_	0.95	0.95	0.05
25 mg	AUC_ss_	0.89	0.89	11.01
	*C*_max,ss_	0.93	0.93	7.22
	*A*_e_	0.72	0.72	0.28
100 mg	AUC_ss_	0.88	0.88	11.81
	*C*_max, ss_	1.01	1.01	0.54
	*A*_e_	0.57	0.57	0.43
Validation dataset: 5 mg				
Fasting healthy single dose	AUC	0.89	1.24	19.52
	*C*_max_	0.74	1.35	25.13
Fed healthy single dose	AUC	1.02	1.21	19.66
	*C*_max_	0.92	1.28	22.86
Fasting healthy multiple doses	AUC	1.15	1.24	25.02
	*C*_max_	0.96	1.28	23.69
	AUC_ss_	0.96	1.21	19.09
	*C*_max,ss_	1.02	1.24	21.03
Fasting T2DM multiple doses	AUC	1.28	1.34	36.08
	*C*_max_	1.14	1.18	18.97
	AUC_ss_	1.05	1.23	22.30
	*C*_max,ss_	1.02	1.15	14.05
Validation dataset: 10 mg				
Fasting healthy single dose	AUC	0.99	1.21	19.20
	*C*_max_	0.93	1.15	12.63
Fed healthy single dose	AUC	1.22	1.29	30.27
	*C*_max_	1.06	1.29	26.54
Fasting healthy multiple doses	AUC_ss (Huang2022)_	1.09	1.22	22.15
	*C*_max,ss (Huang2022)_	1.01	1.14	13.22
	AUC_ss (Que2021)_	1.16	1.24	25.16
	*C*_max,ss (Que2021)_	1.20	1.22	23.38
	AUC_ss (He2023)_	1.00	1.21	19.67
	*C*_max,ss (He2023)_	0.88	1.19	15.45
Fed healthy multiple doses	AUC_ss_	1.11	1.11	11.02
	*C*_max,ss_	0.82	1.21	17.60
Fasting T2DM multiple doses	AUC	1.30	1.35	37.77
	*C*_max_	1.23	1.25	25.72
	AUC_ss_	1.14	1.26	26.55
	*C*_max,ss_	1.18	1.22	22.70
Validation dataset: 20 mg				
Fasting T2DM multiple doses	AUC	1.18	1.28	29.23
	*C*_max_	0.93	1.15	12.96
	AUC_ss_	1.01	1.23	21.09
	*C*_max,ss_	1.07	1.16	15.61
Validation dataset: 25 mg				
Fasting healthy multiple doses	AUC_ss_	0.89	1.12	10.90
	*C*_max,ss_	0.81	1.24	19.35

*Note*: *A*_*e*_, percentage of the administered dose recovered in urine; AUC, area under the time-concentration curve.

Abbreviations: AAFE, absolute average fold error; AAPE, average absolute prediction error; AFE, average fold error; *C*_max_, peak concentration.

^a^The AAFE and AFE were identical in the training datasets because testing was conducted on a single virtual individual rather than a population.

**Table 4:** Prediction performance for henagliflozin PBPK model in hepatic impaired populations.

Group	Parameter	AFE	AAFE	AAPE%
Control 20 mg single dose	AUC_0–72_	1.15	1.26	26.59
	*C*_max_	1.04	1.13	13.06

CP-A: 20 mg single dose	AUC_0–72_	1.01	1.27	24.59
	*C*_max_	0.79	1.29	21.29

CP-B: 20 mg single dose	AUC_0–72_	1.20	1.24	25.43
	*C*_max_	0.90	1.15	12.70

CP-C: 20 mg single dose	AUC_0–72_	1.61	1.61	27.60
	*C*_max_	0.90	1.15	12.47

*Note*: AUC, area under the time–concentration curve; *C*_max_, peak concentration.

Abbreviations: AAFE, absolute average fold error; AAPE, average absolute prediction error; AFE, average fold error.

**Table 5:** Predicted parameters for henagliflozin in pediatric populations.

Group	Regimen	*C*_max,ss_ (ng/mL)	UGE_*C*max_ (g)	AUC_ss_ (ng h/mL)	UGE_AUC_ (g)
Adult (18–60 years)	5 mg once daily	88.09 (14.64)	28.64 (4.76)	592.02 (146.27)	30.10 (7.44)
	10 mg once daily	176.18 (29.28)	36.10 (6.00)	1184.03 (292.54)	37.06 (9.16)

Adolescents (12–18 years)	5 mg once daily	96.27 (17.37)	29.68 (5.36)	670.61 (175.38)	31.49 (8.23)
	10 mg once daily	192.54 (34.74)	36.91 (6.66)	1341.23 (350.76)	38.09 (9.96)

Children (2–11 years)	1.25 mg once daily	47.47 (12.95)	21.16 (5.77)	311.96 (105.63)	22.51 (7.62)
	2.5 mg once daily	94.93 (25.89)	29.52 (8.05)	623.92 (211.26)	30.69 (10.39)
	5 mg once daily	189.87 (51.78)	36.79 (10.03)	1247.84 (422.52)	37.50 (12.70)
	10 mg once daily	379.73 (103.56)	41.95 (11.44)	2495.68 (845.05)	42.18 (14.28)

Infants and toddlers (28 days–23 months)	1.25 mg once daily	123.6 (61.61)	32.50 (16.20)	961.6 (640.45)	35.18 (23.43)
	2.5 mg once daily	247.19 (123.22)	39.01 (19.45)	1923.21 (1280.91)	40.67 (27.09)
	5 mg once daily	494.38 (246.45)	43.36 (21.62)	3846.41 (2561.81)	44.12 (29.38)
	10 mg once daily	988.77 (492.89)	45.92 (22.90)	7692.83 (5123.63)	46.07 (30.68)

Neonates (0–27 days)	1.25 mg once daily	331.47 (65.16)	41.11 (8.08)	2999.04 (919.3)	43.09 (13.21)
	2.5 mg once daily	662.93 (130.32)	44.63 (8.77)	5998.09 (1838.6)	45.50 (13.95)
	5 mg once daily	1325.87 (260.64)	46.62 (9.16)	11,996.18 (3677.2)	46.82 (14.35)
	10 mg once daily	2651.73 (521.27)	47.69 (9.37)	23,992.33 (7354.4)	47.50 (14.56)

*Note*: Parameters are presented as mean values with standard deviations in parentheses. AUC_ss_, area under the time–concentration curve at steady state; *C*_max,ss_, peak concentration at steady state; UGE_AUC_, urinary glucose excretion calculated by AUC-response relationship; UGECmax, urinary glucose excretion calculated by *C*_max_-response relationship.

## Data Availability

Source data and the dataset generated during the study are available from the corresponding authors upon reasonable request.

## Supporting Information

Supporting Information Table S1. The clinical studies used for developing and validating the PBPK model. Table S2. Summary of sensitivity analysis approaches used in the study. Figure S1. Flow chart showing ertugliflozin (A) and henagliflozin (B) metabolism, disposition, and elimination model development strategy. Percentages have been scaled to 100% of the dose compared to reported values in the mass balance study. CLint, intrinsic clearance; CLint,u, unbound intrinsic clearance; CLiv,p, intravenous plasma clearance; CLiv,metab, intravenous metabolic blood clearance; CLr, renal clearance; CYP, cytochrome P450; Fe, fraction excreted; UGT, uridine 5′-diphosphate-glururonosyltransferase. Figure S2. Ontogeny curve for CYP3A4 (duodenum, A), CYP3A4 (liver, B), UGT2B7 (C), and UGT1A9 (D). Figure S3. Concentration-time profiles and goodness of fit for henagliflozin in hepatic impaired populations. (A) Concentration-time profiles of henagliflozin in control and hepatic impaired groups (CP-A, CP-B, and CP-C), showing observed data points and model-predicted curves across various levels of hepatic impairment. (B) GOF plot comparing predicted and observed plasma concentrations for hepatic impaired populations. Solid lines represent the 1.25-FE, while dashed lines indicate the 2-FE boundaries. Figure S4. Sensitivity analysis of henagliflozin across different age groups. Panels (A) and (B) display sensitivity analysis results for adults, while panels (C) and (D) correspond to adolescents aged 12 years. Panels (E) and (F) show sensitivity analysis for children aged 2 years, and panels (G) and (H) represent infants aged 1 month. Each pair of panels provides insights into the impact of various parameters on henagliflozin PKs across these age groups. Figure S5-1. Morris sensitivity analysis of henagliflozin across different pediatric groups. Panel (A) shows results for infants aged 1 month, panel (B) for children aged 2 years, panel (C) for children aged 11 years, and panel (D) for adolescents aged 12 years. Each plot illustrates the relative influence of physiological and drug-specific parameters on PK outputs, with the main effect (μ*⁣*^*∗*^) on the *x*-axis and interaction or nonlinearity (*σ*) on the *y*-axis. Both axes are presented on logarithmic scales. Parameters are color-coded, and the plots highlight age-related differences in dominant factors contributing to henagliflozin exposure. Figure S5-2. EFAST sensitivity analysis of henagliflozin across pediatric age groups. Panels (A) to (D) present the extended Fourier amplitude sensitivity test (EFAST) results for henagliflozin in infants and children at four representative ages: 1 month (A), 2 years (B), 11 years (C), and 12 years (D). Each age group includes two plots: the upper plot shows sensitivity indices for AUC, and the lower for *C*_max_. For each parameter, first-order and total effect indices are shown side-by-side. The analysis highlights both the direct (main effect) and combined (interaction-inclusive) influence of physiological and drug-specific parameters on henagliflozin PKs, with clear shifts in dominant factors across pediatric development.
