# Supplementary material for: Physiologically-Based Pharmacokinetics and Empirical Pharmacodynamic Modeling for Pediatric Henagliflozin Dosing: Clinical Insights for Chinese Patients
Source: Pediatr Diabetes. 2025 Aug 7;2025:8857248. doi: 10.1155/pedi/8857248 (PMC12352996; doi:10.1155/pedi/8857248)
Supplement: Supporting Information — Table S1. The clinical studies used for developing and validating the PBPK model. Table S2. Summary of sensitivity analysis approaches used in the study. Figure S1. Flow chart showing ertugliflozin (A) and henagliflozin (B) metabolism, disposition, and elimination model development strategy. Percentages have been scaled to 100% of the dose compared to reported values in the mass balance study. CLint, intrinsic clearance; CLint,u, unbound intrinsic clearance; CLiv,p, intravenous plasma clearance; CLiv,metab, intravenous metabolic blood clearance; CLr, renal clearance; CYP, cytochrome P450; Fe, fraction excreted; UGT, uridine 5′-diphosphate-glururonosyltransferase. Figure S2. Ontogeny curve for CYP3A4 (duodenum, A), CYP3A4 (liver, B), UGT2B7 (C), and UGT1A9 (D). Figure S3. Concentration-time profiles and goodness of fit for henagliflozin in hepatic impaired populations. (A) Concentration-time profiles of henagliflozin in control and hepatic impaired groups (CP-A, CP-B, and CP-C), showing observed data points and model-predicted curves across various levels of hepatic impairment. (B) GOF plot comparing predicted and observed plasma concentrations for hepatic impaired populations. Solid lines represent the 1.25-FE, while dashed lines indicate the 2-FE boundaries. Figure S4. Sensitivity analysis of henagliflozin across different age groups. Panels (A) and (B) display sensitivity analysis results for adults, while panels (C) and (D) correspond to adolescents aged 12 years. Panels (E) and (F) show sensitivity analysis for children aged 2 years, and panels (G) and (H) represent infants aged 1 month. Each pair of panels provides insights into the impact of various parameters on henagliflozin PKs across these age groups. Figure S5-1. Morris sensitivity analysis of henagliflozin across different pediatric groups. Panel (A) shows results for infants aged 1 month, panel (B) for children aged 2 years, panel (C) for children aged 11 years, and panel (D) for adolescents aged 12 yea [file 8857248.f1.docx]

**Table S1. The clinical studies used for developing and validating PBPK model**

| **Year** | **Race** | **Sample** | **Population** | **Age (years)** | **Treatment** | **Purpose** | **Reference** |
| --- | --- | --- | --- | --- | --- | --- | --- |
| 2021 | Asian | 6 | Fasting Healthy | 25.7 (2.9) | 2.5 mg single dose | Training | Yifan Zhang et al. 2021[1] |
| 2021 | Asian | 10 | Fasting Healthy | 23.5 (2.8) | 5 mg single dose | Training |  |
| 2021 | Asian | 10 | Fasting Healthy | 24.2 (2.8) | 10 mg single dose | Training |  |
| 2021 | Asian | 10 | Fasting Healthy | 25.5 (4.5) | 25 mg single dose | Training |  |
| 2021 | Asian | 10 | Fasting Healthy | 25.8 (3.5) | 50 mg single dose | Training |  |
| 2021 | Asian | 10 | Fasting Healthy | 27.5 (3.8) | 100 mg single dose | Training |  |
| 2021 | Asian | 10 | Fasting Healthy | 22.8 (3.2) | 200 mg single dose | Training |  |
| 2021 | Asian | 6 | Fasting Healthy | 27.0 (4.8) | 1.25 mg once daily for 10 days | Training |  |
| 2021 | Asian | 6 | Fasting Healthy | 25.2 (3.8) | 2.5 mg once daily for 10 days | Training |  |
| 2021 | Asian | 6 | Fasting Healthy | 26.0 (4.0) | 5 mg once daily for 10 days | Training |  |
| 2021 | Asian | 6 | Fasting Healthy | 28.2 (4.0) | 10 mg once daily for 10 days | Training |  |
| 2021 | Asian | 6 | Fasting Healthy | 26.0 (2.4) | 25 mg once daily for 10 days | Training |  |
| 2021 | Asian | 6 | Fasting Healthy | 27.0 (3.5) | 100 mg once daily for 10 days | Training |  |
| 2021 | Asian | 6 | Fasting Healthy | 26.4 (3.06) | 10 mg single dose | Validation | Zhendong Chen et al. 2021[2] |
| 2021 | Asian | 6 | Fed Healthy | 26.7 (2.25) | 10 mg single dose | Validation | Zhendong Chen et al. 2021[2] |
| 2023 | Asian | 17 | Fasting Healthy | 29.0 (7.5) | 5 mg single dose | Validation | Yueyue Liu et al. 2023[3] |
| 2023 | Asian | 17 | Fed Healthy | 29.0 (7.5) | 5 mg single dose | Validation |  |
| 2023 | Asian | 10 | Fed Healthy | 29.0 (7.5) | 5 mg single dose on day 1 and once daily on days 5-11 | Validation |  |
| 2015 | Asian | 8 | Fasting T2DM | 52.4 ± 6.9 | 5 mg single dose on day 1 and once daily on days 4-10 | Validation | Xiaolan Yong et al. 2015 [4] |
| 2015 | Asian | 8 | Fasting T2DM | 50.6 ± 9.1 | 10 mg single dose on day 1 and once daily on days 4-10 | Validation |  |
| 2015 | Asian | 8 | Fasting T2DM | 49.0 ± 6.5 | 20 mg single dose on day 1 and once daily on days 4-10 | Validation |  |
| 2024 | Asian | 12 | Fed Healthy Male | 28.1 (4.34) | 10 mg once daily for 4 days | Validation | Qian Chen et al. 2024 [5] |
| 2015 | Asian | 12 | Fasting Healthy Male | 26 (2.9) | 25 mg once daily for 4 days | Validation | Liupeng Wang et al. 2015 [6] |
| 2022 | Asian | 12 | Fasting Healthy | 20-28 | 10 mg once daily for 4 days | Validation | Yunzhe Huang et al 2022 [7] |
| 2021 | Asian | 12 | Fasting Healthy Male | 29.3 (3.2) | 10 mg once daily for 4 days | Validation | Linling Que et al 2021 [8] |
| 2023 | Asian | 16 | Fasting Healthy Male | 24.3 (3.0) | 10 mg once daily for 4 days | Validation | Xuejun He et al 2023 [9] |
| 2024 | Asian | 8 | Fasting Healthy | 43.9 (5.46) | 20 mg single dose | Validation | Likun Ding et al 2024 [10] |
| 2024 | Asian | 8 | Fasting Mild Hepatic Impairment | 43.6 (7.15) | 20 mg single dose | Validation |  |
| 2024 | Asian | 8 | Fasting Moderate Hepatic Impairment | 41.5 (7.11) | 20 mg single dose | Validation |  |
| 2024 | Asian | 8 | Fasting Severe Hepatic Impairment | 53.1 (12.41) | 20 mg single dose | Validation |  |

1. Zhang Y-f, Liu Y-m, Yu C, Wang Y-t, Zhan Y, Liu H-y, et al. Tolerability, Pharmacokinetic, and Pharmacodynamic Profiles of Henagliflozin, a Novel Selective Inhibitor of Sodium-Glucose Cotransporter 2, in Healthy Subjects Following Single- and Multiple-dose Administration. Clinical Therapeutics. 2021;43(2):396-409. http://doi.org/10.1016/j.clinthera.2020.12.012.

2. Chen Z-d, Chen Q, Zhu Y-t, Zhang Y-f, Zhan Y, Chen X-f, et al. Effects of Food on the Pharmacokinetic Properties and Mass Balance of Henagliflozin in Healthy Male Volunteers. Clinical Therapeutics. 2021;43(9):e264-e73. http://doi.org/10.1016/j.clinthera.2021.07.008.

3. Liu Y, Huyan X, Zhang Q, Qing H, Zhang Q, Wang X, et al. Effects of Food and Multiple‐dose Administration on the Pharmacokinetic Properties of HR20033, a Sustained‐release Formulation of Henagliflozin and Metformin for the Treatment of Diabetes, in Healthy Chinese Volunteers. Clinical Pharmacology in Drug Development. 2022;12(4):376-84. http://doi.org/10.1002/cpdd.1193.

4. Yong X, Wen A, Liu X, Liu H, Liu Y-P, Li N, et al. Pharmacokinetics and Pharmacodynamics of Henagliflozin, a Sodium Glucose Co-Transporter 2 Inhibitor, in Chinese Patients with Type 2 Diabetes Mellitus. Clinical Drug Investigation. 2016;36(3):195-202. http://doi.org/10.1007/s40261-015-0366-7.

5. Chen Q, Yu C, Wu Q, Song R, Liu Y, Feng S, et al. Evaluation of Drug-Drug Interaction Between Henagliflozin and Hydrochlorothiazide in Healthy Chinese Volunteers. Drug Design, Development and Therapy. 2024;Volume 18:1855-64. http://doi.org/10.2147/DDDT.S433377.

6. Wang L, Wu C, Shen L, Liu H, Chen Y, Liu F, et al. Evaluation of drug–drug interaction between henagliflozin, a novel sodium-glucose co-transporter 2 inhibitor, and metformin in healthy Chinese males. Xenobiotica. 2015;46(8):703-8. http://doi.org/10.3109/00498254.2015.1113576.

7. Huang Y, Liu R, Wang Y, Liu G, Wang C, Chen X, et al. Evaluation of Pharmacokinetic Interactions Between the New SGLT2 Inhibitor SHR3824 and Valsartan in Healthy Chinese Volunteers. Clinical Therapeutics. 2022;44(7):945-56. http://doi.org/10.1016/j.clinthera.2022.06.001.

8. Que L, Huang K, Xiang X, Ding Y, Chu N, He Q. No apparent pharmacokinetic interactions were found between henagliflozin: A novel sodium-glucose co-transporter 2 inhibitor and glimepiride in healthy Chinese male subjects. Journal of Clinical Pharmacy and Therapeutics. 2022;47(8):1225-31. http://doi.org/10.1111/jcpt.13659.

9. He X, Liu G, Chen X, Wang Y, Liu R, Wang C, et al. Pharmacokinetic and Pharmacodynamic Interactions Between Henagliflozin, a Novel Selective SGLT-2 Inhibitor, and Warfarin in Healthy Chinese Subjects. Clinical Therapeutics. 2023;45(7):655-61. http://doi.org/10.1016/j.clinthera.2023.06.002.

10. Ding L, Yang L, Ren D, Gao X, Zhang J, Liu M, et al. Pharmacokinetic, Pharmacodynamic, and Safety Profiles of Proline Henagliflozin in Chinese Subjects with Varying Degrees of Liver Dysfunction. The Journal of Clinical Pharmacology. 2024;64(8):1015-22. <http://doi.org/10.1002/jcph.2437>.

**Table S2. Summary of Sensitivity Analysis Approaches Used in the Study**

| Type | Method | Purpose | Key Outputs |
| --- | --- | --- | --- |
| Local Sensitivity Analysis | One-at-a-time (OAT) | Assess influence of individual parameters (e.g., UGT1A9, UGT2B7, GFR) on AUC and Cmax by perturbing one parameter at a time | Sensitivity coefficient (S) |
| Global Sensitivity Analysis | Morris Method | Identify key parameters and assess non-linear or interaction effects (screening purposes) | μ* (mean absolute effect), σ (SD) |
|  | eFAST | Quantify contribution of each input to variance of AUC/Cmax | First-order (Si), Total-order (STi) |

**Local Sensitivity Analysis (LSA):** To evaluate the influence of individual physiological parameters on predicted pharmacokinetic outcomes, a local (one-at-a-time) sensitivity analysis was performed. Each input parameter p was perturbed by ±10% around its baseline value while keeping all other parameters constant. The resulting change in AUC and Cmax was quantified using a normalized sensitivity coefficient:

$$S= \frac{\Delta AUC}{AUC} \cdot\frac{p}{\Delta p} (1)$$

where S is the sensitivity of the PK output (AUC or Cmax) to the parameter, ΔAUC is the change in AUC following perturbation, and Δp is the change in the parameter value. A value of S = 1 implies a 10% change in input yields a 10% change in output. To avoid divergence as Δp approaches zero, each parameter was perturbed by ±10% of its baseline value, in line with commonly accepted PBPK modeling practices.

**Morris Method:** The Morris method is a screening approach used to identify influential parameters while balancing computational efficiency. Elementary effects are calculated by perturbing each parameter across a defined range using a random sampling scheme.

For each parameter, two statistics are computed:

- 𝜇^∗^: the mean of the absolute elementary effects, representing overall influence.
- 𝜎: the standard deviation of the effects, indicating non-linearity and interaction.


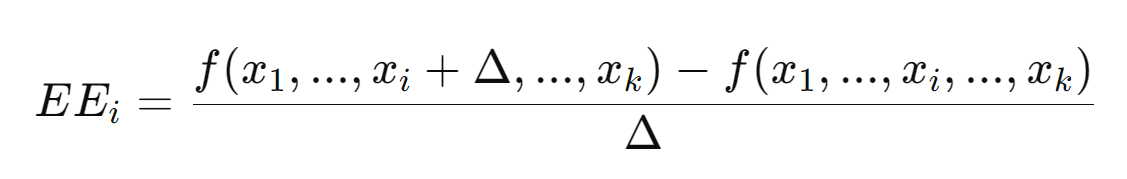


Where Δ is the step size and 𝐸𝐸𝑖 is the elementary effect of the 𝑖-th parameter.

**eFAST Method**: The extended Fourier Amplitude Sensitivity Test (eFAST) decomposes the variance of the output into contributions from each input parameter using frequency domain analysis. It provides:

- First-order sensitivity index 𝑆𝑖, the direct contribution of a parameter,
- Total-order index 𝑆_𝑇𝑖_, capturing all effects including interactions.


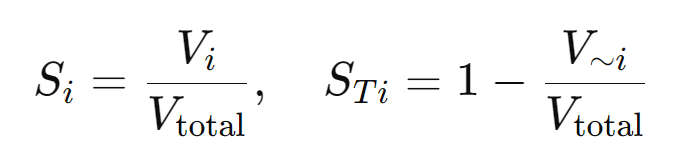


Where 𝑉𝑖 is the variance caused by parameter 𝑖, and 𝑉∼𝑖 is the variance from all other parameters.

Ref: Najjar A, Hamadeh A, Krause S, Schepky A, Edginton A. Global sensitivity analysis of Open Systems Pharmacology Suite physiologically based pharmacokinetic models. CPT Pharmacometrics Syst Pharmacol. 2024;13(12):2052-2067. doi:10.1002/psp4.13256

**
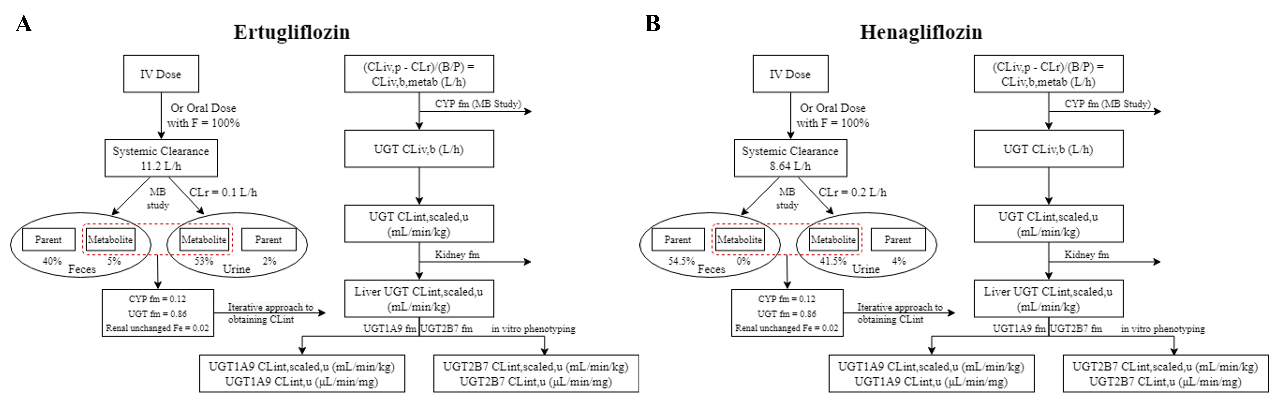
**

**Figure S1**. Flow chart showing ertugliflozin (A) and henagliflozin (B) metabolism, disposition and elimination model development strategy. Percentages have been scaled to 100% of the dose compared to reported values in mass balance study. CLint, intrinsic clearance; CLint,u, unbound intrinsic clearance; CLiv,p, intravenous plasma clearance; CLiv,metab, intravenous metabolic blood clearance; CLr, renal clearance; CYP, cytochrome P450; Fe, fraction excreted; UGT, uridine 5’-diphosphate-glururonosyltransferase.


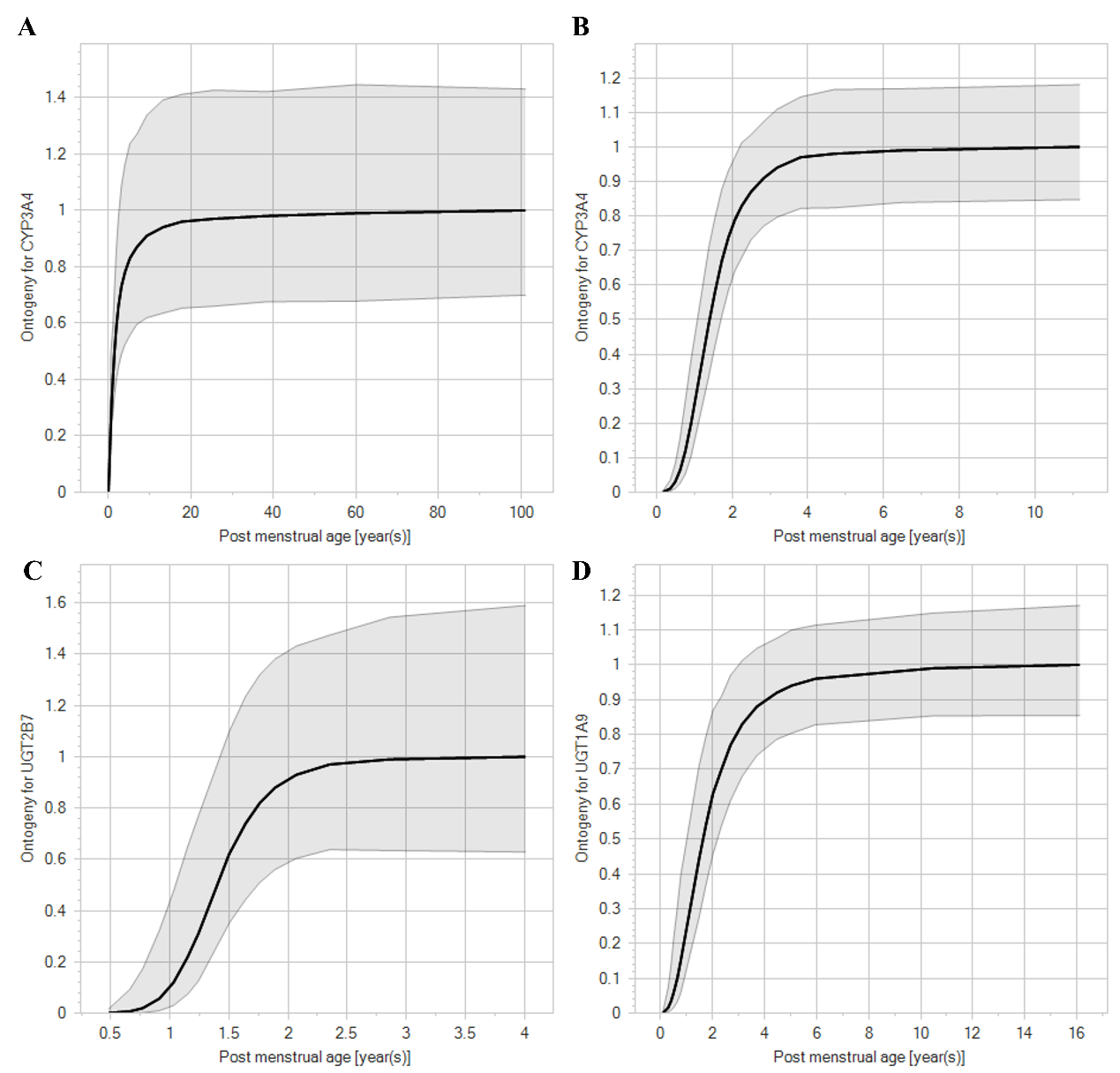


**Figure S2**. Ontogeny curve for CYP3A4 (Duodenum, A), CYP3A4 (Liver, B), UGT2B7 (C), and UGT1A9 (D).


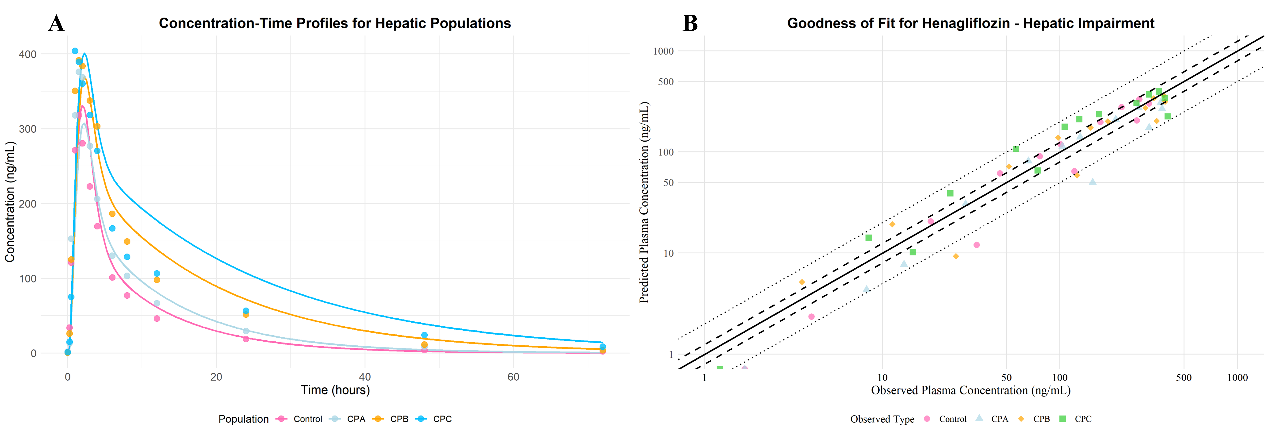


**Figure S3**. Concentration-Time Profiles and Goodness of Fit for Henagliflozin in Hepatic Impaired Populations. (A) Concentration-time profiles of Henagliflozin in control and hepatic impaired groups (CP-A, CP-B, and CP-C), showing observed data points and model-predicted curves across various levels of hepatic impairment. (B) GOF plot comparing predicted and observed plasma concentrations for hepatic impaired populations. Solid lines represent the 1.25-fold error, while dashed lines indicate the 2-fold error boundaries.


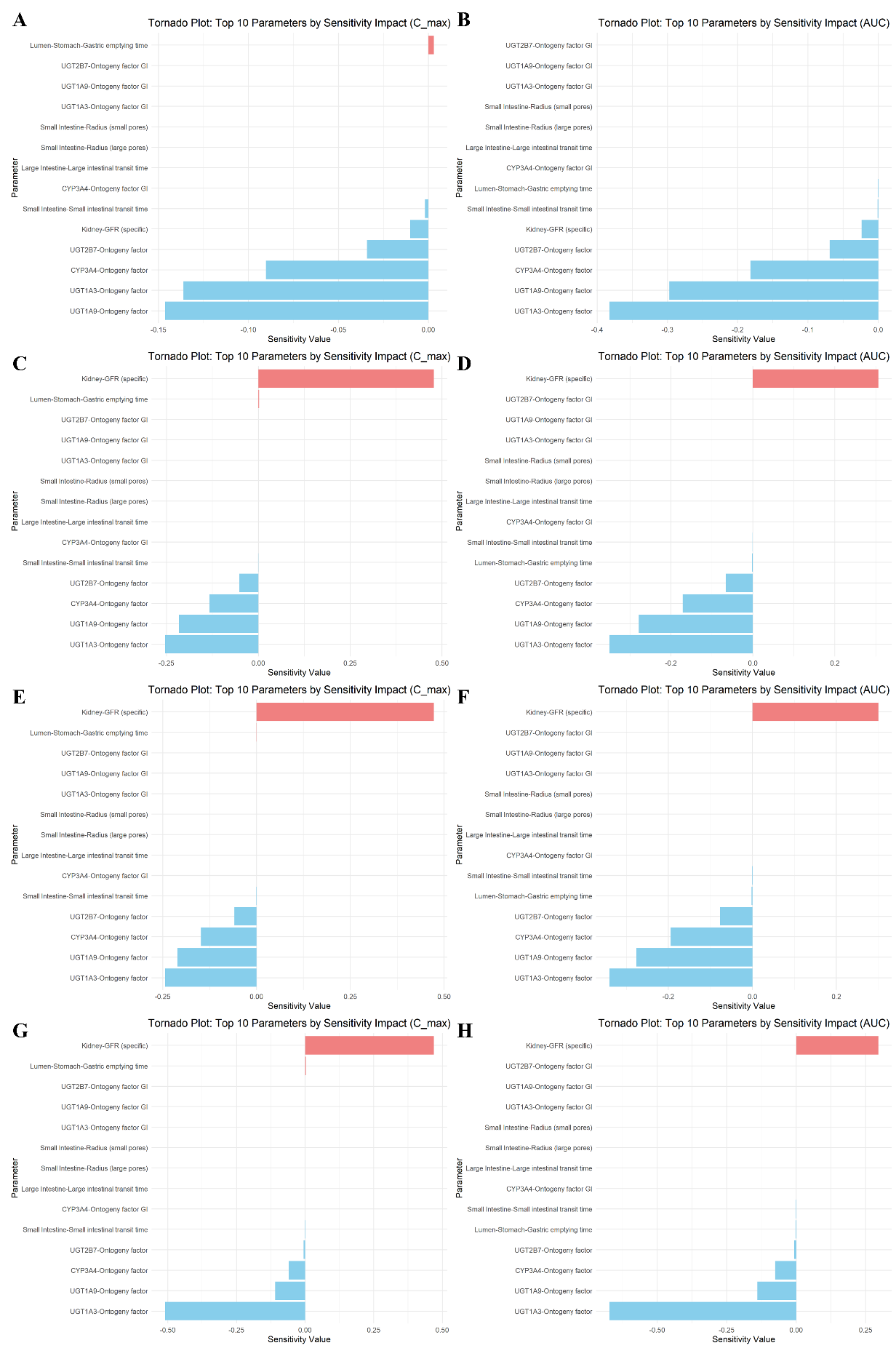


**Figure S4**. Sensitivity Analysis of Henagliflozin Across Different Age Groups. Panels (A) and (B) display sensitivity analysis results for adults, while panels (C) and (D) correspond to adolescents aged 12 years. Panels (E) and (F) show sensitivity analysis for children aged 2 years, and panels (G) and (H) represent infants aged 1 month. Each pair of panels provides insights into the impact of various parameters on Henagliflozin pharmacokinetics across these age groups.


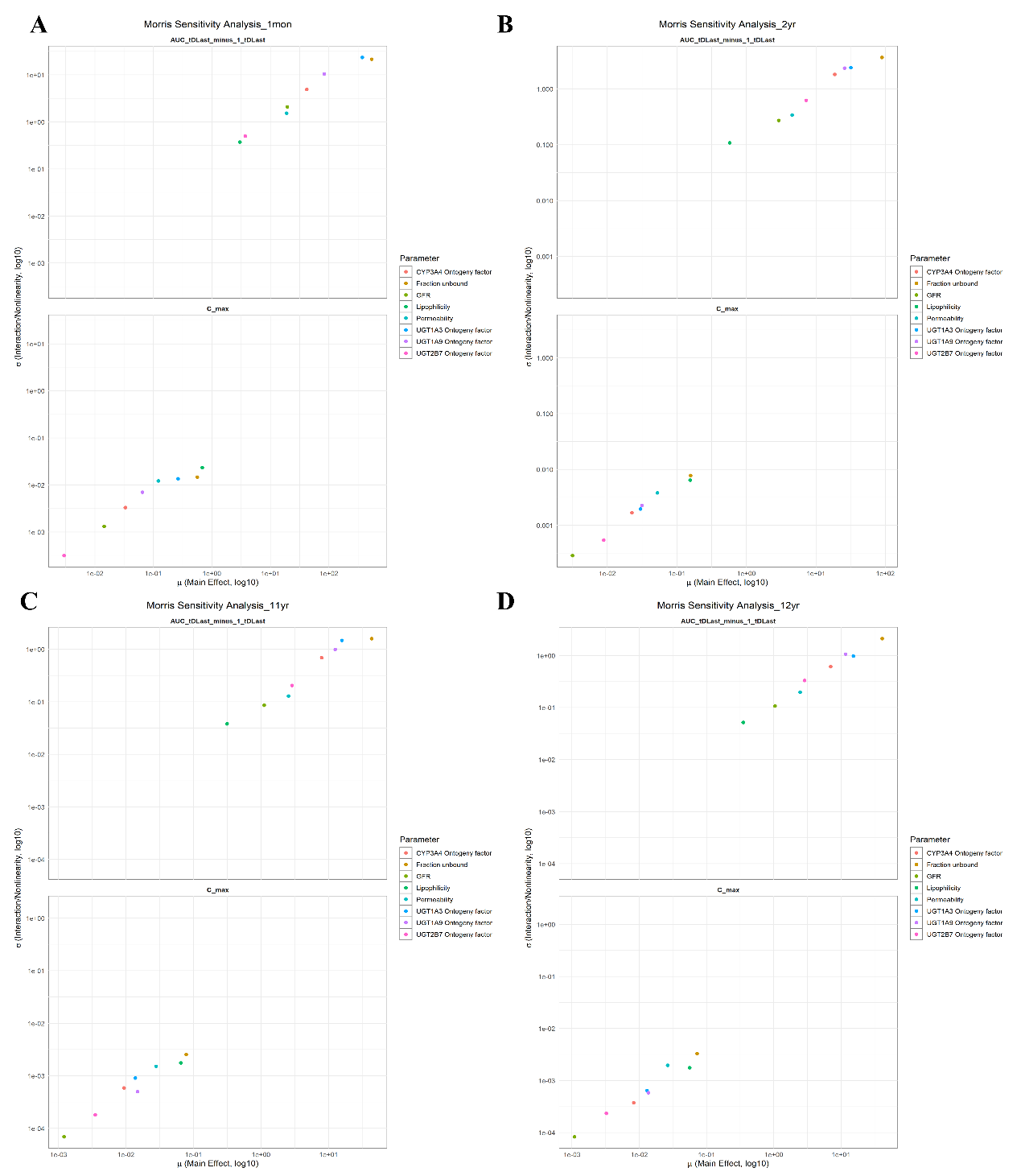


**Figure S5-1**. Morris Sensitivity Analysis of Henagliflozin Across Different Pediatrics Groups. Panel (A) shows results for infants aged 1 month, panel (B) for children aged 2 years, panel (C) for children aged 11 years, and panel (D) for adolescents aged 12 years. Each plot illustrates the relative influence of physiological and drug-specific parameters on pharmacokinetic outputs, with the main effect (μ*) on the x-axis and interaction or nonlinearity (σ) on the y-axis. Both axes are presented on logarithmic scales. Parameters are color-coded, and the plots highlight age-related differences in dominant factors contributing to henagliflozin exposure.


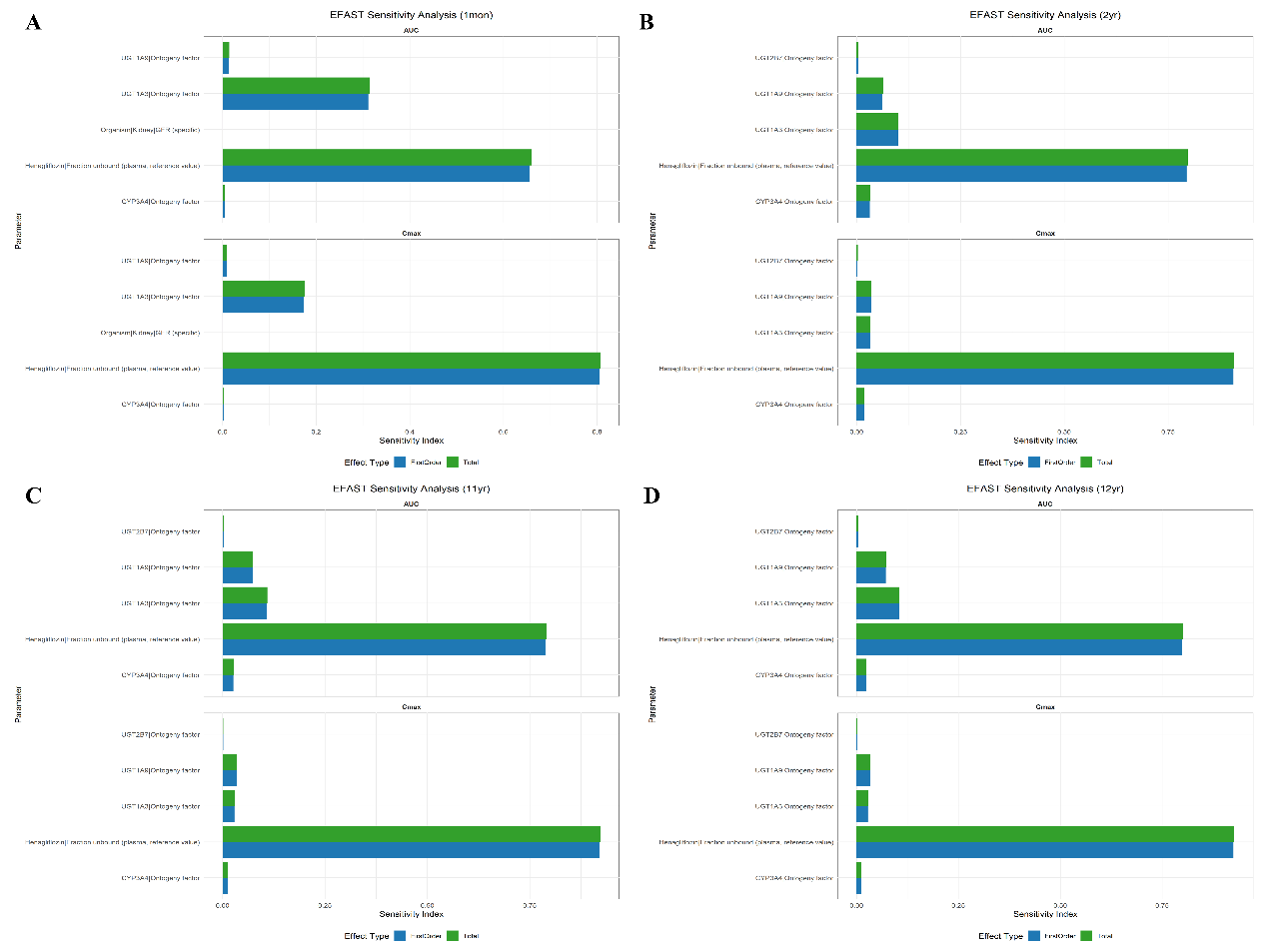


**Figure S5-2**. EFAST Sensitivity Analysis of Henagliflozin Across Pediatric Age Groups.

Panels (A) to (D) present the extended Fourier amplitude sensitivity test (EFAST) results for Henagliflozin in infants and children at four representative ages: 1 month (A), 2 years (B), 11 years (C), and 12 years (D). Each age group includes two plots: the upper plot shows sensitivity indices for AUC, and the lower for Cmax. For each parameter, first-order and total effect indices are shown side-by-side. The analysis highlights both the direct (main effect) and combined (interaction-inclusive) influence of physiological and drug-specific parameters on Henagliflozin pharmacokinetics, with clear shifts in dominant factors across pediatric development.
